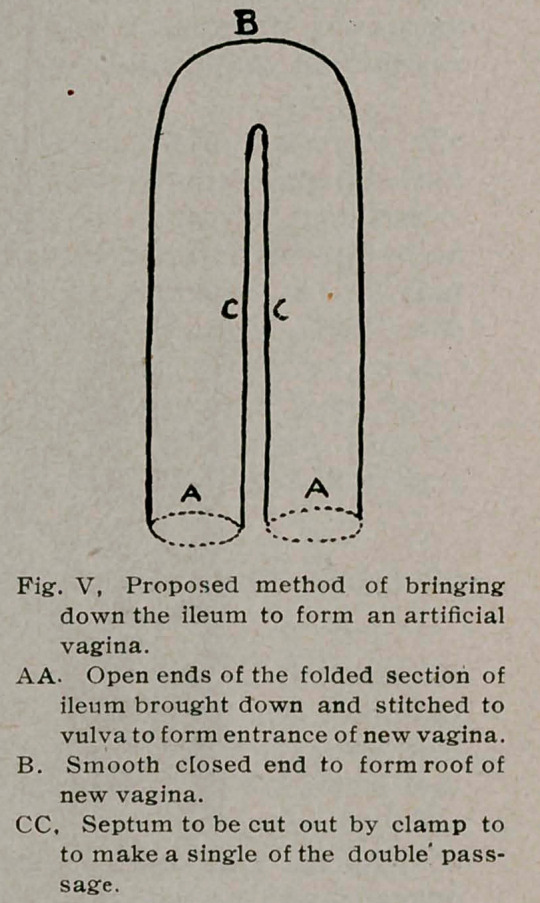# The Construction of a Vagina1Read at the Forty-third annual meeting of the Medical Association of Central New York held at Syracuse, October, 19 and 20, 1910.

**Published:** 1911-02

**Authors:** W. L. Wallace

**Affiliations:** Syracuse, N. Y.


### The Construction of a Vagina.[1]

By W. L. WALLACE, M.D.,
Syracuse, N. Y.

ABSENCE of vagina is acquired or congential. Acquired absence is usually a stenosis, the walls of the vagina being more or less present, even when the passage is entirely closed. It may be caused in utero, the child being born with a vagina closed by a broad adhesion of its mucous membrane; or it may be caused in childhood by scarlet fever or other disease; or in adult life by burns, ulcerations or injuries.

On the other hand, congenital absence of vagina is a defect, caused by the failure to develop of the ducts of Müller, from which tubes, uterus, and vagina are formed. It is usually associated with other anomalies, as rudimentary uterus. The external organs, however, are generally normal, as they are developed from outside structures,—the labia, vulva, clitoris, vestibule, urethra appearing as usual. Ovaries are generally present.

The first operations done for the construction of a vagina were in the cases of stenosed and imperforate vagina, and were for the purpose of evacuating the pent-up blood. They were done through the perineum with trocar, or knife and fingers, and were not usually undertaken until the uterus and tubes were distended with blood. As these operations were usually fatal from infection, many surgeons refused to operate. (See "Construction of a New Vagina," A. Brothers, *American Journal of Obstetrics,* September, 1906.)

If the uterus and tubes are distended, the operation for imperforate vagina or hymen is always very dangerous. In young girls of sixteen or eighteen who have probably menstruated only

---

1. Read at the Forty-third annual meeting of the Medical Association of Central New York held at Syracuse, October, 19 and 20, 1910.

a few times and whose menstrual blood is all contained in the vagina without any distension of the uterus and tubes, it is perfectly safe to cut the hymen, drain off the blood, and put on a weak antiseptic vulvar pack. This I recently did in a newly married woman of eighteen, having first obtained permission to open the abdomen if I should find the uterus and tubes distended.

Fig. I. Rudimentary Uteri ending in small cords which pass behind the bladder (A) in position of absent vagina. Tubes, ovaries and round ligaments normal.

(Mrs. W., Good Shepherd Hospital, Syracuse, N. Y., April 12, 1910.)

In a case of long standing, simple puncture of vaginal stenosis will not be reasonably safe. In such a recent case (Mrs. H., Good Shepherd Hospital, February 22, 1910) in a woman of fifty who had four children and had seen no blood for eight years, I broke through the vaginal stenosis, letting out a large amount of tar-like blood and then, finding the uterus and tubes distended, opened the abdomen and removed them. In this case, the vagina which I thoroughly dilated at the operation has since contracted and again closed, illustrating the fact that all efforts made to maintain the patency of a stenosed vagina by frequent dilatation, as with glass plugs, are fruitless, the sore canal contracting and closing in spite of treatment.

In the next step in the development of an operation for this condition, more successful results were obtained by skin and mucous membrane transplanting and grafting, the grafts being secured from the same or another patient. If the operation is for atresia and the organs above the vagina are in normal condition to secrete, these plastic methods are frequently satisfactory. In congenital cases, however, ordinary plastic operations are not satisfactory. A vagina lined with skin and flaps of mucous membrane, with no uterus about to aid with secretion, is usually dry and tender, and hair may grow from one of the vulvar flaps which has been transplanted within the canal; while contraction is sure to occur.

In 1892 Snegneriff constructed a new vagina out of the lower end of the rectum using the anus for vaginal entrance and making an artificial anus in the sacral region. In 1897 Gersuny split the anus and rectum, leaving a wide strip from the front of the rectum attached to the bladder as the anterior wall of the vagina, which was completed by flaps from the outside. These methods have been imitated by others, but they seem to me very unsatisfactory.

In September, 1904, Dr. James F. Baldwin, of Columbus, O., published in *Annals of Surgery* an operation which he had devised and tried on a cadaver, by which he proposed to utilise a length of sigmoid or ileum as the new vagina, making a resection of the bowel for this purpose through the opened abdomen. In *American Journal of Obstetrics,* November, 1907, Baldwin published a description of his first operation[1], and has given a subsequent report in the *Journal of American Medical Association,* April 23, 1910.

In May, 1906, two years after I had seen Baldwin's first article, and one year before he did his first operation, I had my first case of congenital absence of vagina.

Case I.—Miss E., aet. 19, never menstruated—no vagina, intending to be married. Breasts and external genitals normal. Operation May 11, 1906, at Good Shepherd Hospital, Syracuse, N. Y. Rectal examination under ether: small cord felt in the location of the vagina, branching above to a small mass on each side. The opened abdomen revealed (Fig.1) double rudimentary uteri two and a half inches apart, each half being blunt above and externally. Their size was one inch by one-half by one-half inch, the lower end being continuous with a small cord which was lost behind the bladder. The uteri were connected by a fold of broad ligament, within half an inch of the top of which came the blad-

1. Vide, also, Transactions American Association of Obstetricians and Gynecologists, Vol. XX, 1907.

der. Connected with each uterus was a well-developed round ligament, which tended to pull it close to the internal inguinal ring. A small ovary and fimbriated end of the tube were high on the left; well-developed ovary and outer half of tube on the right. Ovaries were high up at the bifurcation of the common iliac arteries, and the small bodies I had felt in examination were the halves of the uterus. I split the left uterus to see if I could pass a probe into the cord of the vagina and found that the mucous

Fig. II. Sigmoid to be resected.

membrane was only a thread the size of twine. I considered the advisability of grafting a gut to the vulva, but the mesentery of the ileum was not long enough, while the sigmoid was very short, hence I did not think it wise to resect. I therefore dissected between the rectum and bladder and pulled the mucous membrane of the vulva up to the culdesac, splitting the labia minora, making them single instead of double layers, and thereby getting long mucous flaps. This made a vagina of mucous membrane the size and length of two fingers. This girl soon married and has lived happily ever since. This is a good result for a plastic operation, but is poor as compared with the next two cases in which I used an intestine to make the vagina after the method of Baldwin.

Case II.—Miss S., aet. 23. Good Shepherd Hospital, October 8, 1908. History and examination the same as last case. Under ether I dissected through the vulva between the bladder and rectum. The opened abdomen showed the same anatomic conditions as the last case, except that both ovaries were normal in size. I cut out one foot of ileum, leaving it attached to its mesentery, doing a lateral anastomosis with a Murphy button to repair the gut. I then used this foot of ileum to make a vagina. I closed both ends, turning them in, and pulled the middle of the folded gut down to the vulva to which after cutting it open I stitched it with fine silk, thus forming a double vagina. (See Fig. 4.) The Murphy button came away on the tenth day. On this tenth day, under chloroform, I put on a clamp which cut through the septum in five days, making a single vagina of the two arms of the gut. (See *Journal of American Medical Association*, April 23, 1910, for description and drawings of Baldwin.)

Four months after the operation, examination showed the vagina the size and length of two fingers,—moist, smooth, not tender. There was some prolapse of the mucous membrane, which I trimmed under cocaine. Nine months after the operation she was married. Two months later she reported to me that everything was satisfactory to both parties. June 16, 1910, one year after marriage and nearly two years after operation, I examined her at my office. One would not suspect from most rigid examination that the vagina was not absolutely natural. She reported that she was normal in all particulars and that their marriage was entirely happy.

Six months later, in May, 1909, I had the third congenital case, at Good Shepherd Hospital, Syracuse, N. Y.

Case III.—Miss C., aet 19, like the others, had known for some time that something was wrong. The history and examination were the same, and the same anatomic conditions presented when the abdomen was opened. The mesentery of the ileum was too short to come down to the vulva. I, therefore, resected five inches of the sigmoid, getting a mesosigmoid with a good blood supply (Fig. 2) (Fig. 3). I brought one end of the resected piece through the perineum and stitched it to the vulva with silk, turning in the upper end. The Murphy button anastomosis made me anxiety, as the bowels did not move until the tenth day, when the Murphy button came away. She was soon married and her physician, Dr. Williams, of Lafayette, wrote me one year later May 4, 1910: 'Mrs. X was in my office about a month ago. Digital examination of artificial vagina caused no pain and there was no tenderness. Vagina was easily dilatable farther than I could reach. Good mucous discharge. Sexual intercourse perfectly satisfactory to both husband and wife. Bowels normal. Mentally she is much improved and seems perfectly happy.

From my experience in these cases I make the following suggestions:

1. Hereafter in using the ileum for the vagina, instead of bringing down the middle of the gut, stitching it to the vulva and opening it, after the manner of Baldwin (Fig. 4), I should bring down the opened ends of the gut and sew them to the vulva, leaving the closed end of the folded gut to form the roof instead of the entrance to the new vagina (Fig. 5). This would save the necessity of closing and turning in the two ends, which

**Fig. III. Anastomosis complete leaving sigmoid to form vagina.**

procedure takes time and also uses up nearly an inch in length at each end. It also saves the necessity of opening the folded end to sew it to the vulva. Of course while being drawn through the perineum, the ends would be properly protected, as would the open end of the sigmoid. This method would also make a smooth roof for the new vagina and would make the application of the clamp much easier, as the full length of the septum would be cut out, as will be seen in Fig. 5.

2. The intestine should be drawn down until the mesentery is taut, so that the new vagina will not prolapse; and any surplus mucous membrane should be cut away. Of course the mesentery must not be pulled down enough to interfere with the blood supply.

3. I should not again be satisfied with a Murphy button anastomosis of the divided sigmoid, as the feces at this place are too solid to escape easily through so small an opening. I should prefer to tie a very large tube into the proximal end and then slightly invaginate the proximal into the distal portion of the gut, using fine silk to close the joint, and approximate the peritoneum, as suggested by Mayo, and as since used by me in similar cases.

4. In doing such a resection of the sigmoid, one must remember that not only must the anastomosed ends be properly supplied with blood, but the piece of gut used for the vagina must be suitably nourished (Fig. 3). This is a very particular and important matter. The resection of a portion of sigmoid, as for a tumor, where the blood supply of the resected portion does not have to be considered, is not free from danger of interfering with the blood supply of the ends that are left for the anastomosis. When it is also necessary to save a section with good blood supply, the greatest pains must be taken to hold up to the light and study the bloodvessels of the whole mesosigmoid. The importance of making the anastomosis with portions of the sigmoid whose ends may be covered by the mesenteric peritoneum must not be neglected.

CONCLUSION.

While plastic operations are successful in many cases of acquired absence of vagina, in the complete absence of congenital cases, they are far inferior to the wonderful results attained by the intestinal transplantation method of Baldwin.

In regard to the risk of the operation,—in the hands of a surgeon practised in intestinal resection, the danger ought to be extremely little,—no greater than that of the everyday operations which, while perhaps not necessary to save life, are required to make it more pleasant and bearable. At any rate the surgeon will find it absolutely impossible to refuse a young man whose decision to marry is fixed and unshakable, and a young woman who is distracted almost to insanity by finding that she is deformed. Why should he refuse operation, if he has found that it will take away all melancholy and give happiness and satisfaction?

1000 E. GENESEE STREET.

## Figures and Tables

**Fig. I. f1:**
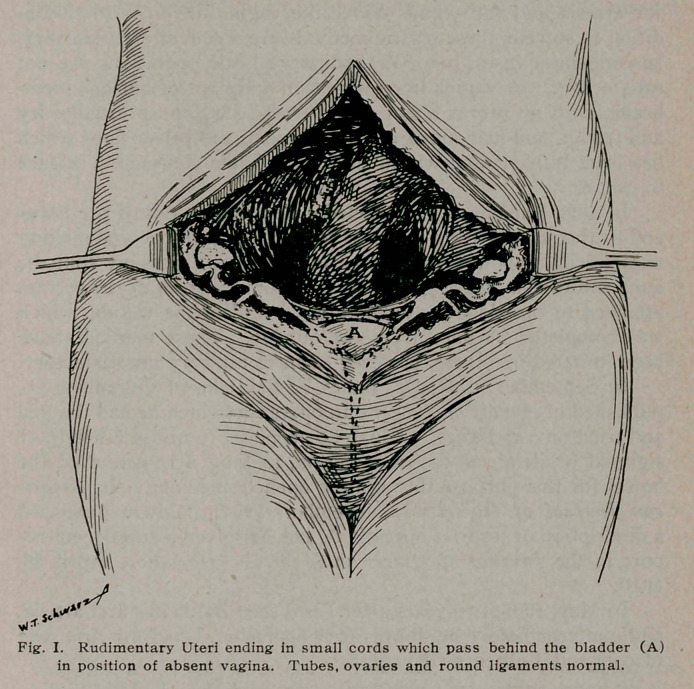


**Fig. II. f2:**
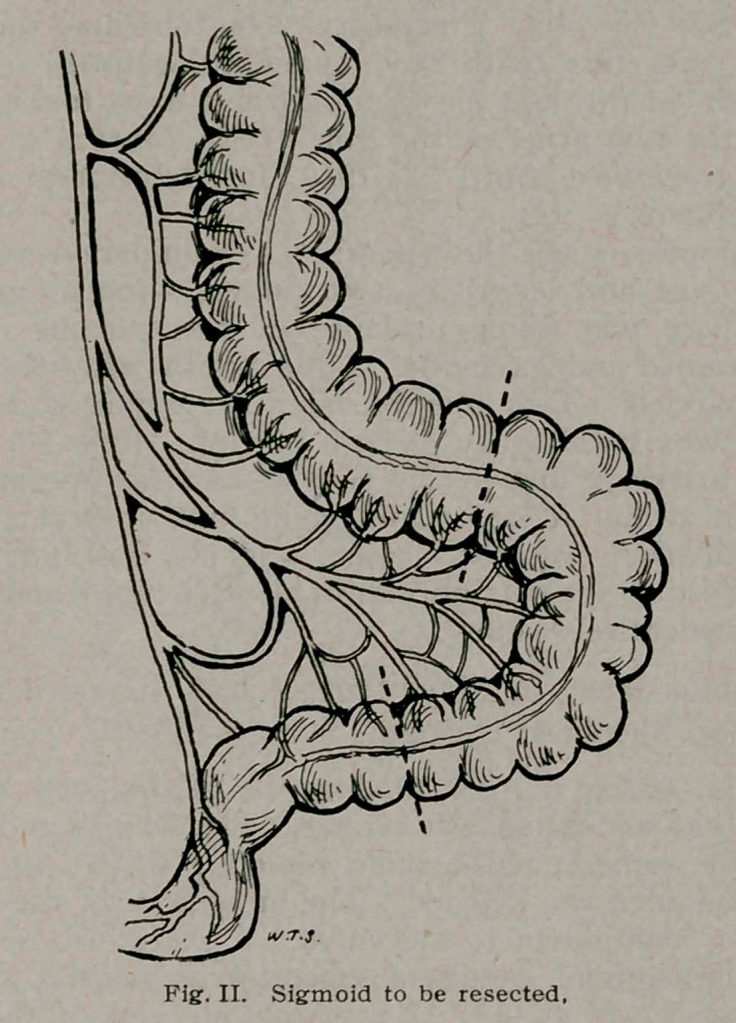


**Fig. III. f3:**
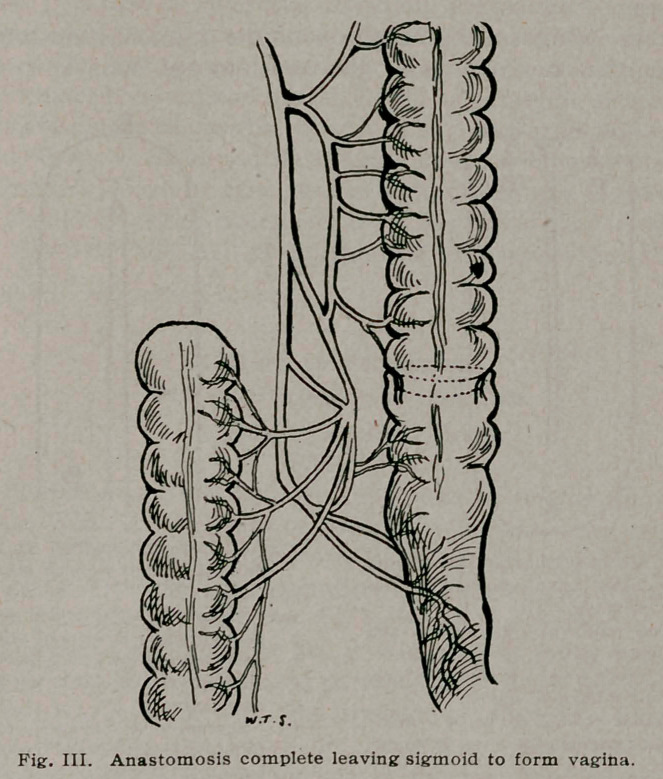


**Fig. IV. f4:**
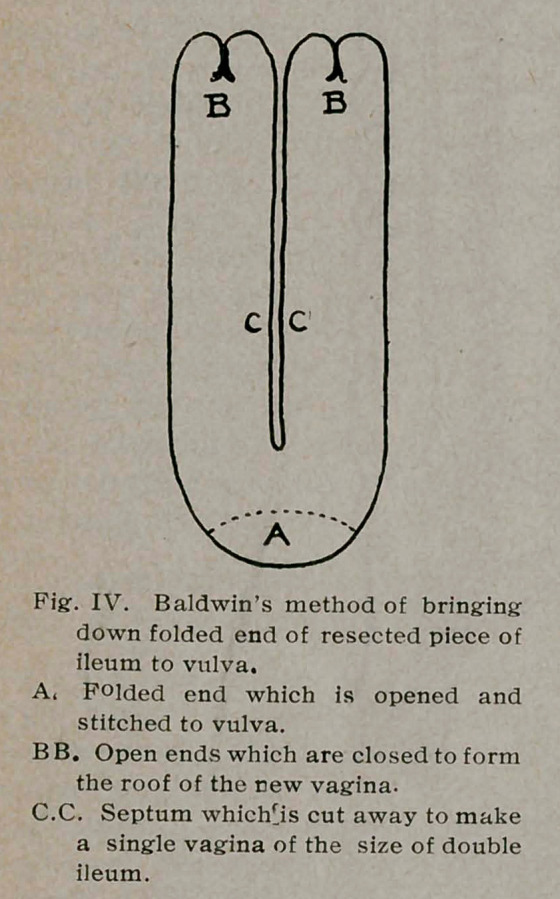


**Fig. V, f5:**